# Out of Sync: A Report of Complete Heart Block

**DOI:** 10.7759/cureus.80851

**Published:** 2025-03-19

**Authors:** Kevin Sande, Oscar Diaz, Guillermo Loyola

**Affiliations:** 1 Internal Medicine, Palmetto General Hospital, Miami, USA; 2 Osteopathic Medicine, Dr. Kiran C. Patel College of Osteopathic Medicine, Nova Southeastern University, Fort Lauderdale, USA; 3 Internal Medicine, University of Florida College of Medicine, Jacksonville, USA

**Keywords:** cardiac pacemaker, complete heart block, idiopathic heart block, third-degree av block, unusual case

## Abstract

Complete heart block is a serious cardiac conduction condition marked by the inability of electrical impulses to propagate from the atria to the ventricles. This disorder causes an absence of coordination between the upper and lower chambers of the heart, resulting in various symptoms and possible problems. Complete heart block, also known as third-degree atrioventricular block, is a more severe form of this illness compared to first and second degree. This report describes a 79-year-old man who experienced a rare presentation of complete heart block. The patient had a preexisting condition of hypertension and had been having episodes of dyspnea for the past few months. Although the patient experienced these symptoms, echocardiography did not reveal any evidence of diminished ejection fraction, indicating that the systolic function was preserved. A left cardiac catheterization procedure was conducted, which showed non-occlusive coronary artery disease. Troponin levels were within normal limits, ruling out an acute ischemic event. This finding eliminates the possibility of severe ischemia factors contributing to the patient's condition. An extensive examination of the patient's medication history showed the absence of any drugs that could have caused the total heart block. The decision was reached to proceed with the implantation of a permanent pacemaker. The pacemaker was effectively inserted, resulting in the elimination of the patient's symptoms and stabilization of his heart rhythm.

## Introduction

Complete atrioventricular block is a rare type of irregular heart rhythm that is becoming more common among elderly individuals in developed countries, likely due to longer lifespans [1]. It is defined by the total inability of electrical impulses to transmit from the atria to the ventricles. The outcome is a notable decrease in heart rate, frequently dropping below 40 beats per minute. While not consistently occurring, syncope caused by idiopathic heart block is also common [2]. This can impair the amount of blood pumped by the heart and result in symptoms such as lightheadedness, exhaustion, fainting, and in severe instances, heart failure or sudden cardiac death.

Primary idiopathic total atrioventricular heart block has been occasionally associated with elevated blood viscosity [3]. This increase in blood viscosity can lead to sluggish blood flow, which may contribute to the disruption of electrical conduction within the heart. In such cases, the atrial rhythm becomes completely autonomous, functioning independently and not influenced by a junctional or lower escape rhythm [4]. This autonomous atrial rhythm can result in a lack of coordination between the atria and ventricles, further complicating the clinical picture. Additionally, external factors such as infections must be considered in the differential diagnosis of heart block. Tick-borne diseases, like Lyme disease, can also potentially lead to the development of total heart block. Lyme carditis, in particular, is a well-documented complication of Lyme disease and can manifest in 1-10% of cases, typically in the second stage of the illness [4]. While many cases of heart block can be associated with structural heart disease and specific medications, there is a significant subgroup of cases where no underlying cause is found, referred to as idiopathic cases. These idiopathic situations might emerge spontaneously without any discernible etiology, making them particularly challenging to diagnose and manage. The therapy of heart block depends on the severity of conduction disruption, and the optimum approach for symptomatic second-degree type II and third-degree heart blocks is the placement of a permanent pacemaker. In order to improve patient outcomes, it is crucial to understand the different categories and associated risks, so that the appropriate therapeutic approach may be determined.

This instance exemplifies an unusual manifestation of total heart block. The absence of drug-related causes and substantial cardiac abnormalities in the patient is remarkable. This example highlights the significance of including total heart block as a potential diagnosis, even when traditional risk markers are not present. It also indicates the necessity for clinicians to be watchful in older individuals who have unclear symptoms, which could be a sign of significant underlying heart issues.

## Case presentation

**Figure 1:**
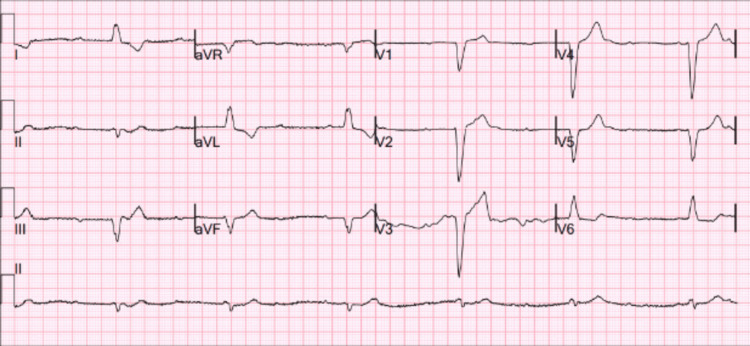
ECG showing complete heart block ECG, electrocardiogram

A 79-year-old male was found by his spouse in a state of extreme drowsiness, exhibiting very low levels of oxygen in his blood and a slow heart rate. Upon reaching the emergency department, the patient's heart rate was measured at 35 beats per minute, and their oxygen saturation was confirmed to be 88% while breathing normal air. An electrocardiogram (ECG) revealed a complete heart block, as shown in Figure 1. A review of the patient's medical records revealed no prior ECGs available for comparison. The absence of documented ECGs made it challenging to determine whether the complete heart block was an acute development or part of a more chronic conduction abnormality. Although the patient experienced a deficient arrhythmic episode, they remained hemodynamically stable. However, they did describe acute weariness, shortness of breath, and generalized body pains.

**Figure 2:**
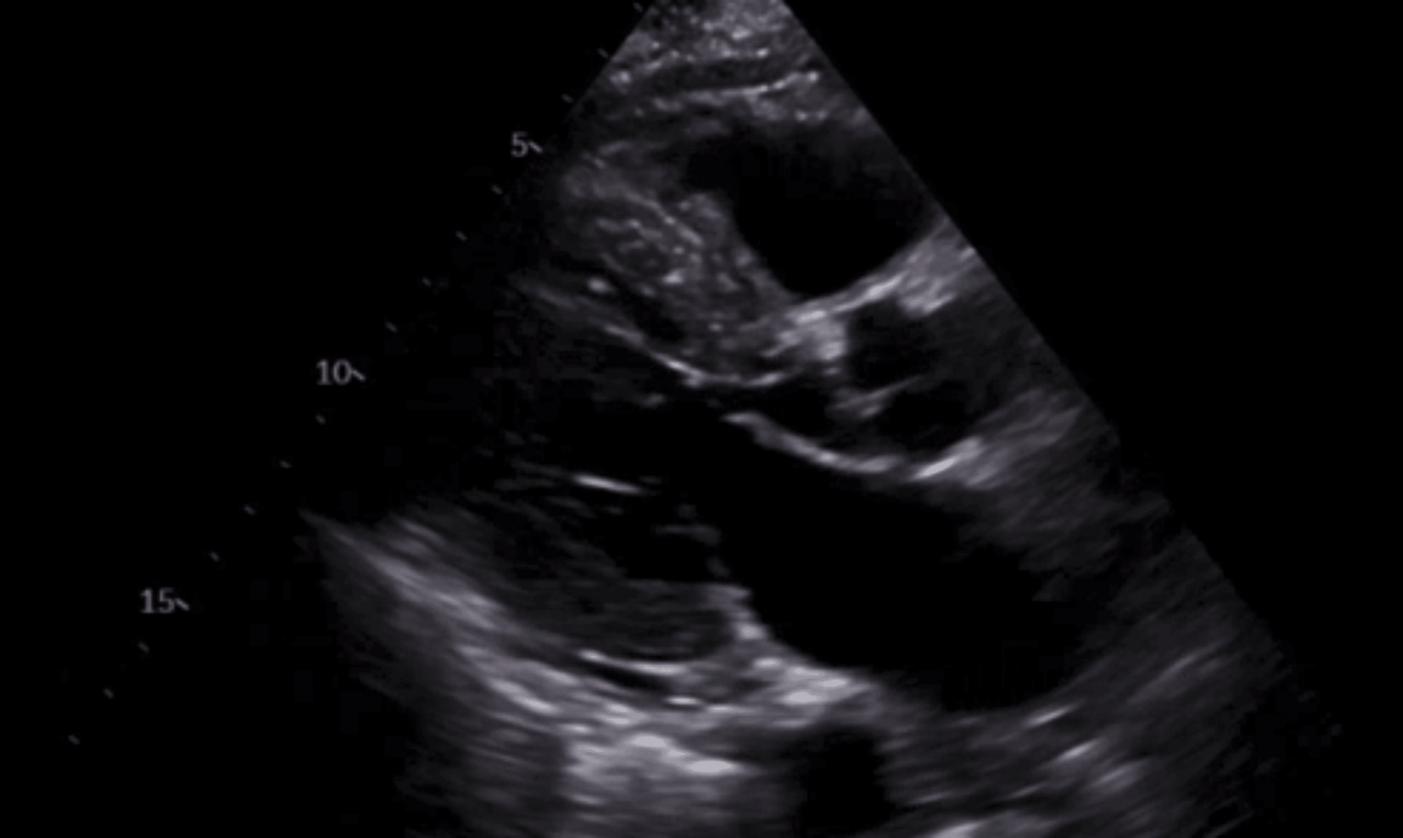
Trans-TTE showing a preserved EF of the left ventricle TTE, thoracic echocardiogram; EF, ejection fraction

Due to the patient's advanced age and the sudden occurrence of total heart block without any prior symptoms or recognized risk factors, a thorough diagnostic investigation was undertaken to identify any underlying causes. The patient had a history of hypertension but no prior cardiac events, structural heart disease, or known arrhythmias. There was no history of obstructive sleep apnea, prior cardiac surgery, or chronic hypoxia-related conditions. These factors were carefully assessed to rule out contributing etiologies. A two-dimensional transthoracic echocardiogram (TTE) with color flow and Doppler was performed, revealing a left ventricular ejection fraction of 45-50%. The study demonstrated moderate concentric left ventricular hypertrophy and severe hypokinesis involving the apical and apical septal walls. Despite these regional wall motion abnormalities, there were no diastolic dysfunction parameters to suggest elevated left atrial pressure or congestive heart failure. The left atrial and right atrial sizes were normal. The mitral valve exhibited mild regurgitation, and there was trace tricuspid regurgitation noted. No pericardial effusion was observed. These echocardiographic findings raised concerns for underlying myocardial disease, although no definitive etiology was identified on imaging as shown in Figure 2. There were no indications of structural heart disease, wall motion abnormalities, or severe valve pathology. Infiltrative cardiomyopathies such as amyloidosis and sarcoidosis were considered, but the absence of left ventricular hypertrophy, restrictive physiology, or echocardiographic signs of infiltration made these conditions unlikely. Additionally, aortic stenosis was ruled out given the lack of valvular calcifications or significant outflow obstruction. These findings provided reassurance by indicating that ischemic cardiomyopathy or significant myocardial dysfunction were not the underlying causes of the heart block.

**Figure 3:**
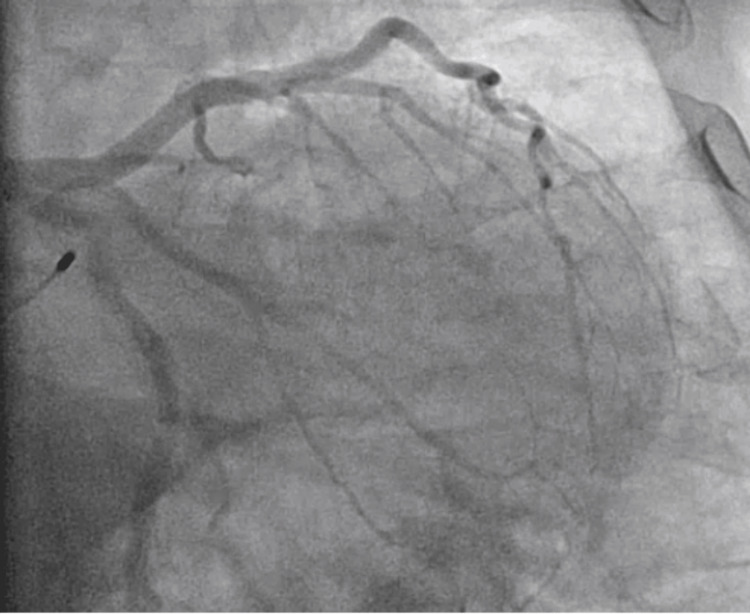
Cardiac catheterization procedure showing non-occlusive disease

In order to further assess the potential presence of ischemic heart disease, the patient underwent coronary angiography as part of the diagnostic evaluation. Left heart catheterization demonstrated that the left coronary artery arises normally, with a large-caliber left main coronary artery bifurcating into the left anterior descending artery (LAD) and circumflex artery, both with non-obstructive disease. The LAD showed no angiographic evidence of obstructive disease and gave rise to three diagonal arteries, all with non-obstructive disease. The circumflex artery also demonstrated luminal irregularities without significant obstruction, with two obtuse marginal branches similarly unaffected by obstructive disease. The right coronary artery was a large-caliber dominant vessel giving rise to a patent posterior descending artery (PDA), with no evidence of obstructive lesions. These findings were consistent with non-obstructive coronary artery disease (NOD), adding complexity to the clinical assessment by failing to reveal a clear ischemic etiology for the complete heart block as shown in Figure 3. While coronary angiography is often performed in conjunction with left heart catheterization, this case focused on imaging the coronary arteries rather than assessing intracardiac pressures. This discovery was not sufficient in describing the source of total heart block, which added more complexity to the clinical situation. The lack of substantial blockage in the coronary arteries or damage to the heart muscle pointed to a non-ischemic cause. However, it is important to acknowledge the possibility of underlying microvascular dysfunction. Microvascular disease, which affects the smaller coronary vessels, cannot be detected by standard coronary angiography and may contribute to myocardial ischemia and conduction abnormalities.

An extensive examination of the patient's medication history was performed to eliminate any potential pharmacological factors contributing to the heart block. The patient was receiving conventional antihypertensive treatment, which included a low-dose ACE inhibitor and a dihydropyridine calcium channel blocker (amlodipine). Dihydropyridine calcium channel blockers primarily affect vascular smooth muscle, causing vasodilation, and are not typically associated with AV conduction abnormalities. Importantly, the patient was not on non-dihydropyridine calcium channel blockers such as verapamil or diltiazem, which are more likely to impair AV node conduction and potentially contribute to heart block. There had been no recent alterations in medication, and the patient had no previous record of using anti-arrhythmic medications, digitalis, or beta-blockers that might have reasonably caused the conduction abnormality. The patient's laboratory blood electrolytes, thyroid function, and inflammation markers were within the normal range. These results did not provide any new information on the cause of the heart block.

**Figure 4:**
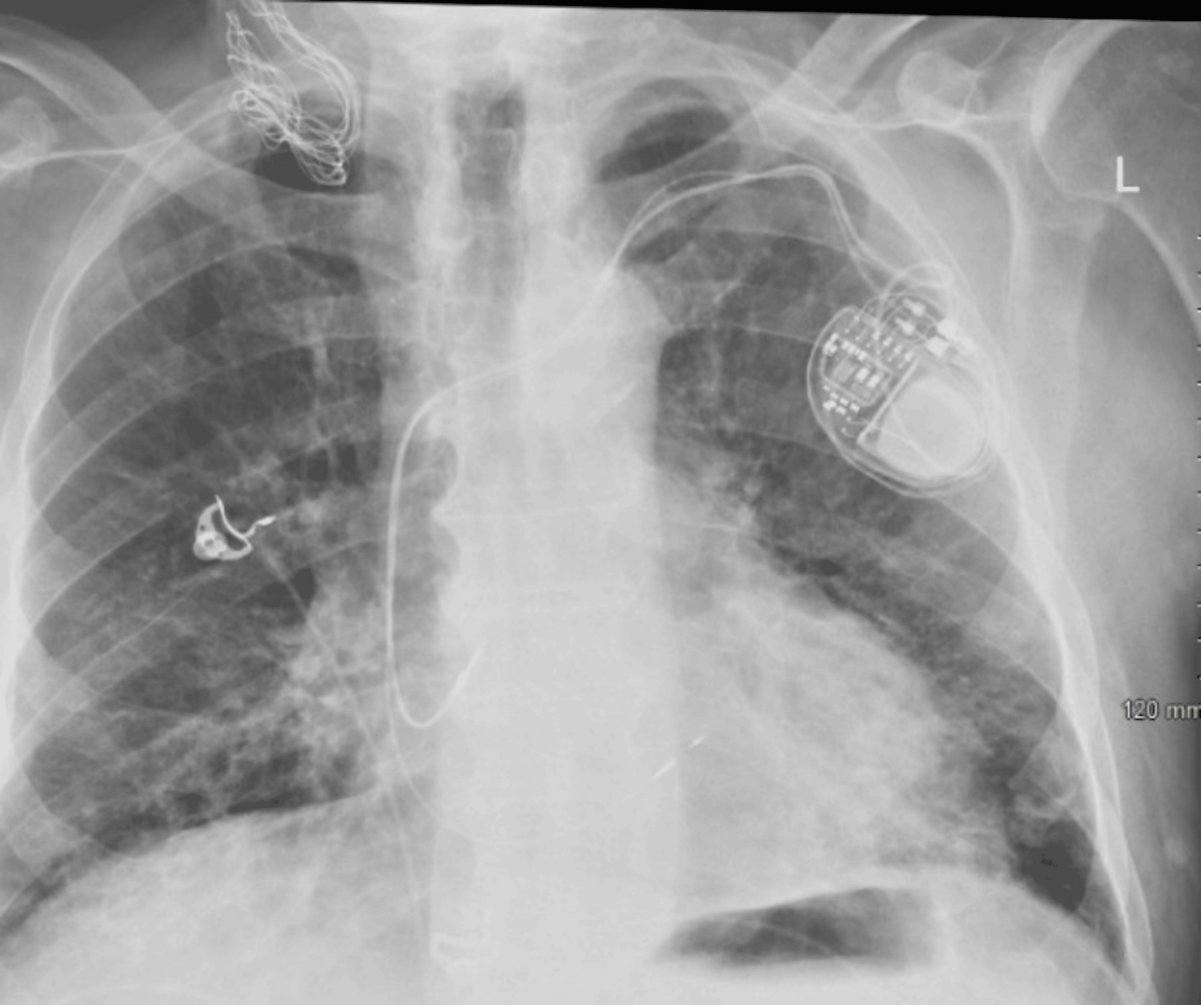
Chest X-ray showing dual-chamber pacemaker placement

Due to the lack of recognizable risk factors or precipitating situations and the seriousness of the conduction abnormality, it was decided to proceed with the implantation of a permanent pacemaker. The patient underwent the procedure without any adverse events, and following the surgery, there was a significant improvement in his symptoms. The pacemaker effectively normalized the heart rate, resulting in the alleviation of his dyspnea, weariness, and myalgia. The patient underwent implantation of a Meditech Azure XT DR MRI Surescan dual-chamber pacemaker (DDD mode, rate set from 60 to 130 bpm), with the ventricular lead placed in the right ventricular apex. This device was chosen for its MRI compatibility and rate-adaptive pacing capability. The pacemaker was implanted on the right side, as confirmed by a post-procedural chest X-ray in Figure 4.

**Figure 5:**
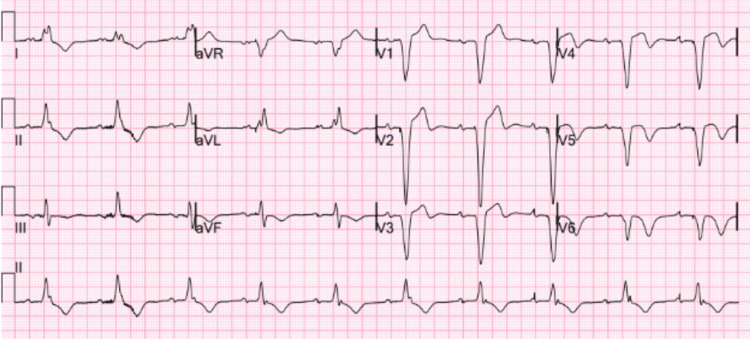
ECG after permanent pacemaker placement ECG, electrocardiogram

This placement was chosen for its simplicity, reliability, and well-established use in managing third-degree atrioventricular block. Other suitable conduction system pacing techniques such as left bundle branch area pacing or his bundle pacing were considered but not utilized in this case. The decision was influenced by the patient's preserved left ventricular ejection fraction, the absence of structural heart disease or pre-existing ventricular dyssynchrony, and the technical challenges and resource requirements associated with conduction system pacing. The ECG after the pacemaker placement is displayed in Figure 5. The successful clinical stability achieved with the insertion of a pacemaker emphasizes the significance of early intervention in instances of total cardiac block, even when the underlying etiology is unknown.

## Discussion

While a temporary pacemaker is often used initially to stabilize patients with complete heart block while investigating potentially reversible causes, in this case, the decision was made to proceed directly with a permanent pacemaker due to the absence of any reversible etiology and persistent symptoms. This emphasizes the importance of individualized patient management based on thorough clinical assessment. It is primarily linked to cardiac problems that are underlying, such as ischemic heart disease, cardiomyopathies, or degenerative illnesses of the conduction system. Complete heart block often occurs due to recognized risk factors such as suboptimal blood pressure and fasting glucose levels [5]. In order to prevent severe complications such as fainting, heart failure, or sudden cardiac death, the condition usually needs prompt intervention, commonly in the form of a permanent pacemaker installation. Only 19.1% of patients diagnosed with total heart block are capable of achieving a full recovery after being admitted to the hospital [6].

In the instance discussed in this case, the patient exhibited an atypical occurrence of total heart block, without any known risk factors or triggering situations. Although the patient is elderly, which is known to increase the risk of conduction disorders, there is no record of any significant coronary artery disease, heart attack, or structural heart disease. This is supported by the preserved ejection fraction observed on echocardiography and the absence of any blockages found on coronary angiography. Furthermore, a thorough examination of his medication records indicated that he did not utilize any drugs that are known to impact the AV node or contribute to the occurrence of heart block. This case is particularly noteworthy since it defies the current understanding of the causes of total heart block, given it lacks the usual risk factors associated with this condition.

The diagnostic findings in this case emphasize its unusual characteristics. The presence of intact ejection fraction and non-occlusive coronary artery disease effectively eliminated ischemic cardiomyopathy or severe myocardial damage as probable explanations. In addition, the patient's laboratory workup ruled out other common secondary causes of total heart block, such as electrolyte abnormalities, thyroid problems, or inflammatory signs. The most likely explanation for this patient's condition is idiopathic degeneration of the cardiac conduction system. However, it is worth noting that this condition usually has a more gradual onset, unlike the acute presentation observed in this case.

This example emphasizes the significance of taking into account idiopathic total heart block in elderly individuals, especially in the absence of traditional risk factors. The patient's symptoms were effectively resolved when the pacemaker was implanted, which highlights the need to promptly identify and address such cases. Furthermore, it prompts significant inquiries on the fundamental pathophysiology of total heart block in patients who do not exhibit apparent triggering circumstances. This case enhances the overall comprehension of heart block, namely by emphasizing that idiopathic cases can develop even without severe co-morbidities, and emphasizes the crucial importance of pacemaker therapy in efficiently managing these individuals.

## Conclusions

Idiopathic complete heart block in an elderly patient without typical risk factors serves as a reminder of the importance of maintaining a high index of suspicion for severe conduction disorders, even in the absence of common precipitating factors. Clinicians should consider idiopathic causes in cases even when traditional risk factors are absent and ensure prompt evaluation and intervention, such as pacemaker implantation, to prevent potentially life-threatening complications. This case also highlights the need for further research into the underlying mechanisms of idiopathic heart block, particularly in the elderly, to improve diagnostic and therapeutic strategies for uncommon condition presentation.
